# The response to oestrogen deprivation of the cartilage collagen degradation marker, CTX-II, is unique compared with other markers of collagen turnover

**DOI:** 10.1186/ar2596

**Published:** 2009-01-20

**Authors:** Anne-Christine Bay-Jensen, Nadine CB Tabassi, Lene V Sondergaard, Thomas L Andersen, Frederik Dagnaes-Hansen, Patrick Garnero, Moustapha Kassem, Jean-Marie Delaissé

**Affiliations:** 1 Department of Clinical Cell Biology, IRS/CSFU, University of Southern Denmark, Vejle Hospital, Kabbeltoft 25, 7100 Vejle, Denmark; 2 Department of Biomarkers, Synarc, 16, rue Montbrillant, Buroparc T4, 69416 LYON cedex 03, France; 3 Institute of Human Genetics, University of Aarhus, Wilhelm Meyers Allé, build. 1240, 8000 Århus C, Denmark; 4 Department of Microbiology and immunology, University of Aarhus, Wilhelm Meyers Allé, build. 1240, 8000 Århus C, Denmark; 5 Department of Clinical Endocrinology and Molecular Biology, University of Southern, Winsloev Parken 25, 5000 Odense C, Denmark

## Abstract

**Introduction:**

The urinary level of the type II collagen degradation marker CTX-II is increased in postmenopausal women and in ovariectomised rats, suggesting that oestrogen deprivation induces cartilage breakdown. Here we investigate whether this response to oestrogen is also true for other type II collagen turnover markers known to be affected in osteoarthritis, and whether it relates to its presence in specific areas of cartilage tissue.

**Methods:**

The type II collagen degradation markers CTX-II and Helix-II were measured in the body fluids of premenopausal and postmenopausal women and in those of ovariectomised rats receiving oestrogen or not. Levels of PIIANP, a marker of type II collagen synthesis, were also measured in rats. Rat knee cartilage was analysed for immunoreactivity of CTX-II and PIIANP and for type II collagen expression.

**Results:**

As expected, urinary levels of CTX-II are significantly increased in postmenopausal women and also in oestrogen-deprived rats, although only transiently. However, in neither case were these elevations paralleled by a significant increase of Helix-II levels and PIIANP levels did not change at any time. CTX-II immunoreactivity and collagen expression were detected in different cartilage areas. The upper zone is the area where CTX-II immunoreactivity and collagen expression best reflected the differences in urinary levels of CTX-II measured in response to oestrogen. However, correlations between urinary levels of CTX-II and tissue immunostainings in individual rats were not statistically significant.

**Conclusions:**

We found only a small effect of oestrogen deprivation on cartilage. It was detected by CTX-II, but not by other type II collagen turnover markers typically affected in osteoarthritis.

## Introduction

The molecular mechanism of osteoarthritis (OA) development is poorly understood. Cartilage alterations in the joint start very locally, extend progressively and lead to inflammation [1]. Several studies have suggested that changes in the cartilage occur well before damage to the cartilage matrix can be detected, and that they are related to modifications in the metabolism of type II collagen and proteoglycans [2-5]. The trigger switching the chondrocyte to a pathological state has, however, not been identified.

OA has multiple aetiologies, but is most often believed to result from mechanical injuries. There are also suggestions that oestrogen deprivation favours OA development [6]. This hypothesis was first suggested by epidemiological studies showing that menopause coincides with the appearance of many of the symptoms associated with OA (i.e. marked incidence of knee OA at menopause compared with men of similar age), and that hormone replacement therapy influences the disease activity [7-10]. The hypothesis was also supported by the fact that chondrocytes have oestrogen receptors [11,12], and that long-term oestrogen replacement therapy has a chondroprotective effect in monkeys [13]. Recently, strong support for this hypothesis came from the development of diagnostic tools to allow monitoring of cartilage degradation in a dynamic way. Thus, it was found that menopause coincides with an increase in the urinary levels of CTX-II, a fragment of type II collagen originating from its telopeptide region, and that this increase correlates with joint damage and is antagonised by oestrogen [14-16]. In order to experimentally test the hypothesis that oestrogen deprivation may favour OA development, rat ovariectomy experiments were performed. They showed that ovariectomy increases the levels of CTX-II in urine, and may induce mild lesions in the articular cartilage [17].

However, despite all these data, the relevance of CTX-II to cartilage turnover in post-menopausal-like situations has not been definitively demonstrated, and a number of questions still need to be answered. The ovariectomy-induced increase in CTX-II level in the rat experiments is transient: it occurs two to four weeks after ovariectomy and decreases after about six weeks. This transitory increase in CTX-II contrasts with the permanent ovariectomy-induced increase in CTX-I, a type I collagen degradation marker reflecting bone resorption [17]. At first sight, this observation would mean that the effect of oestrogen deprivation is permanent on bone, but not on cartilage; however, the reason for this difference is unclear.

It is surprising that elevated levels of CTX-II drop not only in response to oestrogen and related agents, but also in response to bone resorption inhibitors not expected to affect cartilage, such as bisphosphonates [18,19]. Presently, CTX-II is the only cartilage degradation marker that has been investigated in response to oestrogen deprivation, and it would therefore be interesting to investigate whether oestrogen deprivation similarly affects other type II collagen degradation markers, such as Helix-II, which corresponds to a fragment originating from the helicoidal part of type II collagen [20].

The interest of comparing CTX-II and Helix-II is also stressed by the fact that, despite both being elevated in OA patients, their levels in the body fluids do not correlate strictly with each other, and their immunoreactivity distributes differently across different histological areas of OA knees [20,21]. This difference suggests that the markers may reflect different collagenolytic pathways, which possibly respond differently to oestrogens. Presently, the effect of oestrogen deprivation on CTX-II is based essentially on assessment of its urinary levels, and it has not been systematically analysed if these urinary levels reflect the local cartilage events where CTX-II originates from. Cartilage sections have indeed been examined only at late time points when CTX-II levels of ovariectomy-rats were back to the control levels of sham-operated rats.

The present study aims to investigate the relevance of CTX-II to cartilage collagen metabolism in situations of oestrogen deprivations, and addresses therefore several of the above questions. First it extends CTX-II to Helix-II measurements both in pre- and post-menopausal women and in rats after ovariectomy treated or not with oestrogen. It also investigates whether these collagen degradation products may relate to the breakdown of newly synthesised collagen, because collagen synthesis is reported to be upregulated in OA [22-24]. PIIANP, the fetal propeptide, appeared to be an especially relevant marker of OA [22,25]. Second, our present study extends body fluid measurements of CTX-II to immunostainings of CTX-II in knee cartilage of ovariectomised rats, by analysing cartilage sections from ovariectomised rats at early time points where CTX-II is increased compared with sham-operated rats. Whether oestrogen deprivation induces lesions in the cartilage, as well as collagen synthesis is also examined.

## Materials and methods

### Healthy premenopausal and postmenopausal women

Fifty healthy premenopausal women (age 30 to 40 years, mean age 35 years) and 50 healthy untreated postmenopausal women (age 48 to 73 years, mean age 59 years) were included in the study. None of the patients had symptomatic OA, and this was confirmed by WOMAC index (Western Ontario and McMaster Universities index of arthritis) and radiography. Serum samples from a biobank in Lyon, where all participants had signed an informed consent allowing the use for scientific purposes, were used. The use of the biobank was approved by local French authorities for the use of biomarker measurement. All premenopausal women had regular cyclic menses. All postmenopausal women had been amenorrhoeic for at least five years. All pre- and postmenopausal women were healthy with no disease or treatment that may interfere with bone and cartilage metabolism including oestrogen replacement therapy in postmenopausal women. For all women a fasting serum sample collected before 9 am and a second morning void urine sample were collected and stored below -70°C until ready for assay for urinary CTX-II and urinary Helix-II.

### Ovariectomy rat model design

The rat ovariectomy protocol was approved by the Danish Experimental Animal Inspectorate under the Ministry of justice (jour. no. 2003/561-795). Sixty acclimated, female virgin, seven-month-old Sprague-Dawley rats (Charles River Laboratory, Kisslegg, Germary) were maintained under standard conditions of 12-hour day and night cycles. Rats were given common chow (Altromin 1314, Brogaarden A/S, Denmark) and water *ad libitum*. Three to four rats were kept together in cases and cared for daily by an animal technician. Rats of seven months of age were used to reduce the release of CTX-II from the growth plate into the body fluids as much as possible [17]. Rats were then randomised to two equal size groups assigned to a two-week (A) and six-week (B) experiment. Rats of these two groups were further divided randomly into three subgroups: eight rats for sham operation (Sham), 11 rats for ovariectomy plus oestradiol (OVX+oestradiol) and 11 rats for ovariectomy plus placebo (OVX+placebo), giving a total of 30 rats in each group.

Rats were premedicated with 5 mg/kg midazolam (Dormicum, Hameln pharmaceuticals, Hameln, Germany) subcutaneously and anaesthetised with 4% isoflurane (Abbott, Gentofte, Denmark) in air. After surgery the animals were given 0.05 mg/kg buprenophin (Temgesic, Schering-Plough A/S, Ballerup, Denmark) subcutaneously and this dose was also given twice a day for two days.

Ovariectomy was performed using a dorsal midline incision and the entrance into the abdominal cavity was made with a small cut in to the muscle half to two-thirds of the way down the side of the rat. The ovaries were pulled out through the muscle incision by grasping the periovarian fat, and the ovaries were removed with a single cut. The uterus was returned into the abdominal cavity. The skin incision was sutured using absorbable sutures.

**Figure 1:**
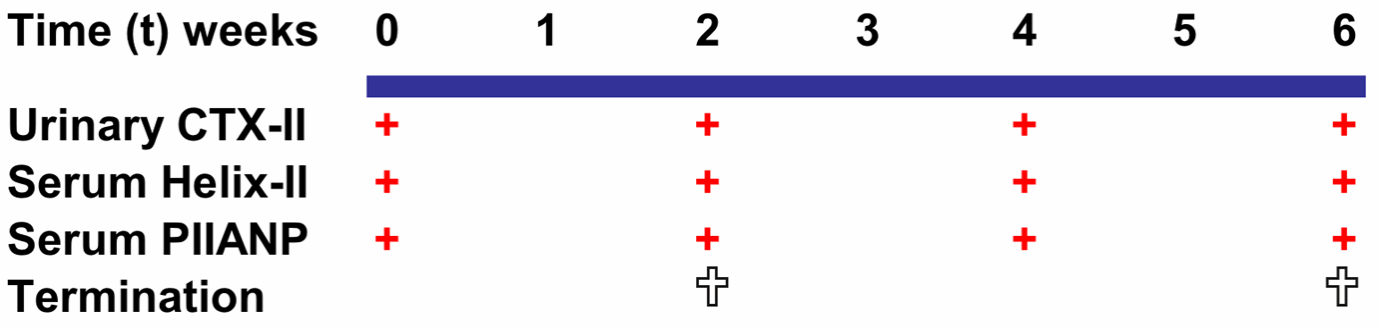
Experimental flow diagram of the ovariectomy rat experiment. Blood and urine samples were isolated at day of ovariectomy (t = 0 weeks) and at two, four and six weeks after ovariectomy. Group A was terminated two weeks post-ovariectomy and group B six weeks post-ovariectomy.

At surgery, a 60-day slow release pellet containing either placebo or 0.05 mg 17α-ethylenestradiol (Innovative Res. of America, Sarasota, FL, USA) was implanted subcutaneously. Four rats died prematurely immediately after ovariectomy: from group A, one OVX+oestradiol and two OVX+placebo rats; from group B, one OVX+oestradiol rat. An additional OVX+placebo rat was excluded from group A, because it failed to urinate at given time points. Final numbers of rats included in the study are given in Table 1. Urine and serum samples were collected before surgery (baseline, t = 0 weeks) and thereafter every second week (Figure 1). Urine collection was achieved by spotting for up to two hours in clean grid bottom cages and serum was taken from ocular blood (retro-orbital; Figure 1). The body weight (Table 1) and health status of the rats were recorded every week. Group A and B rats were euthanased after two (group A) and six weeks (group B), respectively.

**Table 1:** Body and uterus weight of ovariectomised rats at baseline and at endpoint

			Bodyweight (g)			Uterus weight (mg)
				
Grp	Subgrp	n	t = 0	t = 2	t = 6	Endpoint
A	Sham	8	307.6 ± 37.2	303.0 ± 31.4		943.9 ± 247.3
	OVX+ oestradiol	10	336.8 ± 32.6	282.0 ± 21.7*		1033.2 ± 288.2
	OVX+ placebo	8	327.1 ± 30.6	340.0 ± 21.2		386.6 ± 100.7***

B	Sham	8	323.4 ± 38.1	318.0 ± 33.6	318.8 ± 36.8	692.2 ± 151.7
	OVX+ oestradiol	10	315.4 ± 20.6	270.5 ± 11.3***	286.6 ± 10.5*	699.0 ± 133.3
	OVX+ placebo	11	337.7 ± 30.4	351.2 ± 33.1*	373.3 ± 40.3**	287.3 ± 174.4***

Values are shown as mean ± SD. Significant levels calculated by Mann-Whitney U tests; * p < 0.05, ** p < 0.01 and *** p < 0.001. Time (t) is in weeks.

The left hind leg from each rat were fixed in formalin for 48 hours at room temperature, and decalcified in 7% idranal (Riedel-van Häen, Sigma-Aldrich, Glostrup, Denmark) for three to four weeks depending on individual knee. Decalcified knees were cleaved into about two sections using the medial collateral ligament as a guide. These two pieces were paraffin-embedded, and then sectioned parallel to this cleavage plane until the central area of the medial tibia plateau was reached, as previous studies showed the prevailing interest of this area [17,26].

### Measurement of biomarkers CTX-II, PIIANP and Helix-II in body fluids

Urinary CTX-II was measured with a competitive ELISA (CartiLaps, IDS Nordic, Denmark) based on a mouse monoclonal antibody raised against the EKGPDP sequence of human and rat type II collagen C-telopeptide. This sequence is specific for the C-telopeptide of type II collagen. Intra- and inter-assay CVs (coefficient of variation) were lower than 8% and 15%, respectively [15]. CTX-II measurements were corrected for the urinary creatinine level and measured by a colorimetric assay [27]. Rat serum CTX-II could not be measured in the current study. Serum PIIANP was measured by an ELISA [23] using polyclonal antibodies raised against recombinant GST-human type II procollagen exon 2 fusion protein [28], and which cross-react with rat type II procollagen. It was not possible to measure PIIANP because of the lack of human serum. Helix-II was measured by a competitive ELISA (Syncart, Synarc, Lyon, France) based on a rabbit polyclonal antibody raised against the amino acid 622–632 sequence of the α1 chain of human and rat type II collagen. Intra- and inter-assay variations (CVs) were lower than 9% and 14%, respectively [20]. Serum levels of Helix-II were measured in rats, and urinary levels were measured in humans, because of the limited sample availability of human serum. Raw data from individual rats at two (group A), four and six (group B) weeks were baseline-corrected by subtracting baseline (time 0) followed by division by the baseline values, and finally multiplying by 100% to give the percentage difference from baseline.

### Histopathological assessment of the rat knees

Previous studies have shown that ovariectomy-induced alterations in rat knee cartilage mainly affected the medial tibia plateau [17,26], so we obtained two 5 μm sections representative of this area, that were 80 to 100 μm apart. These sections were mounted on positive loaded glass slides (Superfrost plus, Hounisen, Denmark). Sections were then deparaffinised and rehydrated. One slide was taken for histology and several slides were used for immunohistochemistry. Slides for histology were stained with a standard Fast green-Safranin O trichrome protocol [29]. A modified Colombo score was used to assess the degree of possible damage where scores of 0, 1 or 2 was given for the 10 different pathological features: loss of superficial layer; erosion; fibrillation; cyst; osteophyte; loss of proteoglycan; disorganisation of chondrocytes; clonal chondrocytes; exposure of subchondral bone; subchondral vascularisation. Colombo score normally consists of scores 0 to 4, but our previous experience with the ovariectomised rat model at the given time points led us to simplify the scoring system, so that only scores 0, 1 and 2 were given, and defined as follows: 0 = the feature was not observed; 1 = the feature was observed, but was weak; 2 = the feature was pronounced and well-defined. The sections were analysed blindly.

### Immunohistochemistry

Rehydrated sections, adjacent to the ones used for histological analysis, were demasked in Target retrieval buffer^® ^(Dako, Glostrup, Denmark) at a pH of 6.0 overnight at 63°C. Sections were then incubated with a peroxidaxe blocking reagent^® ^(Dako, Glostrup, Denmark) for 10 minutes and with 0.5% Casein (Sigma-Aldrich, Denmark) in Tris-buffered saline (TBS) for 20 minutes, both at room temperature. After blocking, sections were incubated with either rabbit anti CTX-II (1:3000), rabbit anti PIIANP (1:1500) or their respective preimmune sera overnight at 4°C. Antibodies used have previous been described [21]. Of note was that PIIANP recognises the N-terminal pro-peptide of type IIA collagen both as part of the proprotein and in its cleaved form [22]. Bound antibodies were then cross bound to the polymer reagent Envision^+ ^anti-rabbit-HRP^® ^(Dako, Glostrup, Denmark) for 30 minutes at room temperature. Immunoreactivity was visualised by DAB^+ ^reagent^® ^(Dako, Glostrup, Denmark).

Finally, sections were counter stained with Mayers acidic haematoxylin for 12 seconds, dehydrated and mounted with pertex. Sections were rinsed carefully between each step with TBS. This protocol is the result of an optimisation, as several alternatives to each step have been thoroughly tested. The Helix-II antibodies proved to be inappropriate for immunohistochemistry on cartilage section from rats.

### *In situ *hybridisation

A 261 bp cDNA fragment (bp 215-476, [Genbank:L48440]) of rat procollagen type IIα1 (exon 1) was synthesised with flanking promoter regions for RNA polymerases T3 and T7 and cloned into at pU57 cloning vector (GenScript, NJ, USA). The plasmids containing the cDNA were linearised, and this served as a template for the *in vitro *transcription of antisense and sense riboprobes labelled with [α-^33^]-UTP (GE Healthcare, Broendby, Denmark). Probes were DNAse treated and purified on a G50 column. *In situ *hybridisation was performed on 5 μm section of the decalcified and paraffin-embedded knees, using a previously described procedure [30]. Briefly, the sections were digested in proteinase K, acetylated and incubated with the riboprobes overnight at 55°C. The sections were then treated with RNAse A and washed extensively. They were then coated with LM-1 auto-radiographic emulsion (GE Healthcare, Broendby, Denmark), exposed for up to four weeks and, developed and counterstained with H&E.

### Statistics

The body and uterus weight at different time points were compared with a one-way analysis of variance (ANOVA). The differences between biomarkers measured in pre- and postmenopausal biomarkers were analysed by Mann-Whitney U tests. The difference in a given biomarker at time point t = a and time point t = 0 (baseline) was calculated in percent as follows:

(X_t = a _- X_t = 0_)/X_t = 0 _* 100%,

where X is the measure obtained for the corresponding marker.

The differences between treatments were analysed with a one-way ANOVA (Turkey). Correlations between markers were calculated by linear regression and values of Pearson's correlation coefficient (r^2^) and the likelihood of non-zero slope (p) is stated. Differences in Colombo score were compared by Mann-Whitney U statistics. The relative frequency of positive events by *in situ *hybridisation or immunohistochemistry in a given experimental group was calculated by adding the number of samples showing positive stainings at a specific zone, and dividing this sum by the total number of samples in this experimental group, to obtain a value of between 0 and 1. Correlations between the level of serum/urinary markers and *in situ *hybridisation/immunohistochemistry were analysed by Mann-Whitney U tests. For all statistical analysis, p < 0.05 was considered significant: *p < 0.05, **p < 0.01 and ***p < 0.001.

## Results

### Measurement of CTX-II, Helix-II in pre- and postmenopausal women

**Figure 2:**
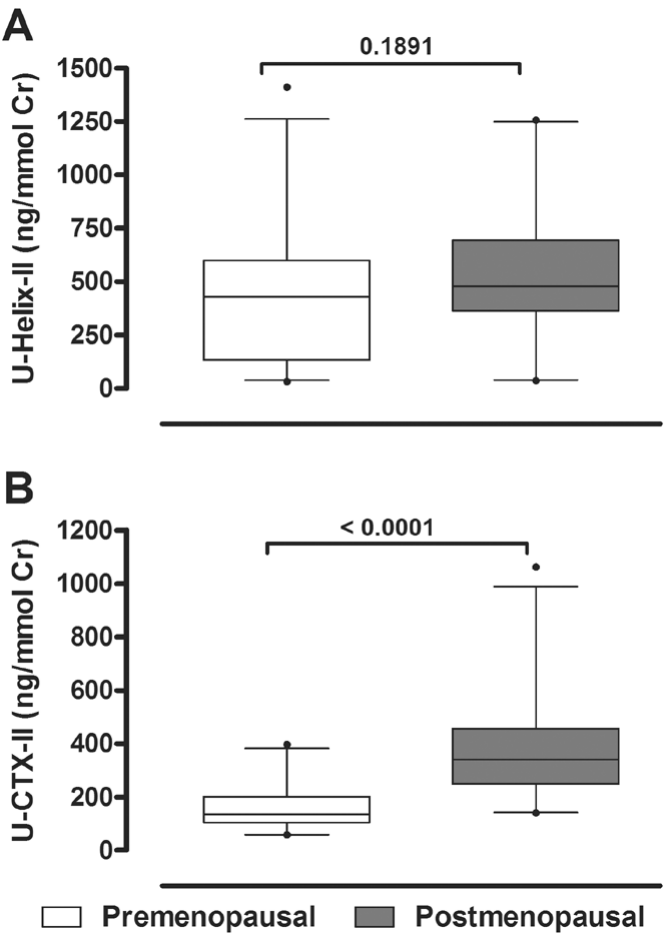
Measurement of urinary levels of **(a)** Helix-II and **(b)** CTX-II in pre- and post-menopausal women. The values are corrected for creatinine. n = 50 in the respective populations. Bars indicate the 95% range and outliers are shown as dots. The difference between the two populations was compared by Mann-Whitney U tests.

Until now CTX-II was the only cartilage degradation marker shown to be elevated in postmenopausal women. It was therefore interesting to investigate whether Helix-II would also be elevated in postmenopausal women, and to what extent Helix-II values would correlate with CTX-II. By assaying urine from 50 premenopausal and 50 postmenopausal women for the degradation biomarkers Helix-II and CTX-II, we found that only CTX-II was significantly increased in postmenopausal women (Figure 2). Helix-II remained unchanged, and there was no correlation between the two markers (r^2 ^= 0.044, p = 0.663). Thus, we reproduced the CTX-II response to menopause seen by others [15,26], but did not find any indication for a response of the other type II collagen degradation marker, Helix-II.

### Changes in body and uterus weight of the ovariectomised rats

To investigate the initial effects of oestrogen deficiency on cartilage in a more controlled way than in postmenopausal women, we used an ovariectomy rat model, which has been previously described as a model of postmenopausal OA [17,26,31]. To determine the efficiency of the ovariectomy, the uterus of all euthanased rats was weighed: all OVX+placebo rats, in both groups A and B, had a significantly lower uterus weight after two and six weeks. Furthermore, all OVX+oestradiol rats had a high uterus weight, which indicated that the oestradiol implants had the desired compensatory effect (Table 1). As expected, ovariectomy (OVX+placebo) induced weight gain, whereas ovariectomised rats with oestradiol implants (OVX+oestradiol) had a loss of body weight two to six weeks after ovariectomy (Table 1). The sham-operated rats did not show any change in weight through the two to six weeks post-ovariectomy. In order to investigate whether ovariectomy-induced weight gain was associated with oestradiol production, we measured serum oestradiol levels at different times post-ovariectomy, but most samples showed values below the detection limit (data not shown).

### The serum or urinary levels of Helix-II, CTX-II and PIIANP in ovariectomised rats

**Figure 3:**
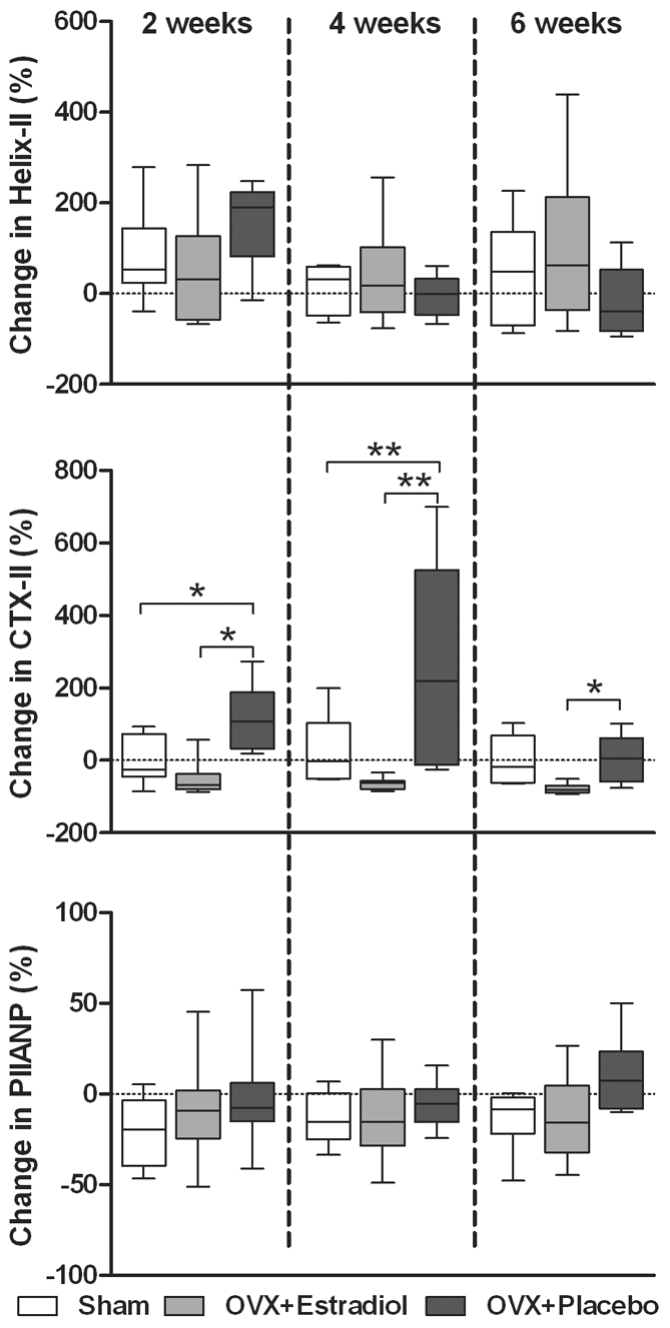
Measurement of type II collagen markers Helix-II, CTX-II, and PIIANP in ovariectomised rats. The percentage difference from baseline value (time (t) = 0) is shown for each rat treated with sham (white boxes), OVX+oestradiol (grey boxes), OVX+placebo (black boxes). **(a)** Serum levels of Helix-II; **(b)** urinary levels of CTX-II; **(c)** serum levels of PIIANP. Bars represent median values. The mean (standard deviation) baseline levels of Helix-II, CTX-II and PIIANP were 8.69 (6.48) ng/ml, 1675 (636.4) μg/mmol creatinine and 324.0 (141.4) ng/ml, respectively. The 95% range is indicated by the bars. Changes induced as a function of time were compared with each other and treatments were compared by one-way analysis of variance, where *p < 0.05 and ** p < 0.01.

We measured the levels of type II collagen degradation markers, serum Helix-II and urinary CTX-II, at time 0 and week 2 in group A, and at time 0, and weeks 4 and 6 of group B. We found a significantly increased level of CTX-II after two and four weeks in the OVX+placebo subgroups but not in the Sham and OVX+oestradiol groups. This increased level drops to baseline six weeks after ovariectomy (Figure 3b). The difference between OVX+placebo and OVX+oestradiol is still significant after six weeks (Figure 3b). Taken together, these data show that we could reproduce the CTX-II profiles obtained previously [17]: that CTX-II is significantly elevated two and four weeks after ovariectomy, but decreases to baseline after six weeks, and is maintained at a low level if the ovariectomy rats receive oestrogen. Interestingly, in contrast to CTX-II, Helix-II levels did not change significantly with time in response to ovariectomy, and were not statistically different from the Helix-II levels in the Sham and OVX+placebo subgroups (Figure 3b). Neither was there any correlation between CTX-II and Helix-II when the complete set of samples taken at the different time points was analysed (r^2 ^= 0.016, p = 0.344).

Next, we examined whether the increased CTX-II levels relate to an increased type II collagen synthesis in the rats and therefore measured the synthesis marker PIIANP. Serum levels for PIIANP did not change significantly over the six-week time course: its levels were more or less the same throughout the weeks and irrespectively of treatment (Figure 3c). There are no correlations between the levels of urinary CTX-II or serum Helix-II and serum PIIANP (r^2 ^= 0.043, P = 0.117). Our rat ovariectomy experiments therefore show that oestrogen deprivation transiently affects CTX-II levels as shown previously, but did not provide evidence for an effect on Helix-II and PIIANP.

### Histopathological approaches

We examined whether increased levels of CTX-II in body fluids are reflected at the level of the knee joint. First, we analysed the medial tibia plateau and the surrounding areas two and six weeks after ovariectomy, because this was the prevailing area showing alterations nine weeks after ovariectomy [17,26]. At these earlier time points of the present experiment, however, only mild alterations such as ulceration of the superficial surface, loss of superficial layers, proteoglycan loss and cluster formation were observed. More rats showed the latter two features at the six-week time point, but there was no significant difference between Sham, OVX+oestradiol or OVX+placebo rats. We concluded that there is no apparent effect of ovariectomy on histology six weeks post-surgery.

**Figure 4:**
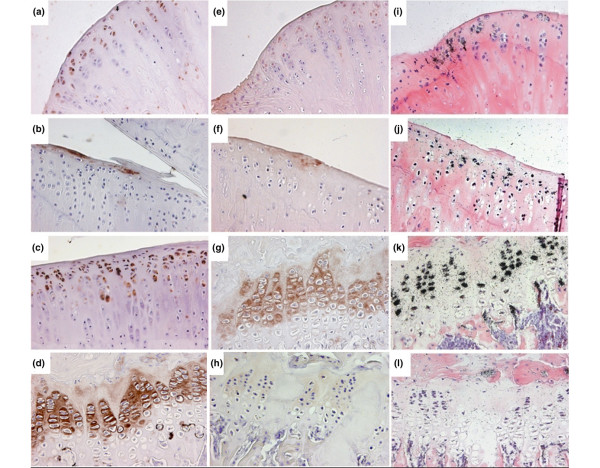
Illustrative examples of immunostainigs of CTX-II and PIIANP and *in situ *hybridisations of type IIA collagen mRNA. All immunohistochemistry sections were stained with DAB+ (brown) and counterstained with Mayers acidic haematoxylin (blue). *In situ *hybridisations were developed with silver grains (black) and counterstained with H&E staining. CTX-II immunoreactivity was observed **(a)** around chondrocytes at the inner zone, **(b)** at superficial matrix of the upper zone, **(c)** around the round and flat chondrocytes of the upper and deep zone and **(d)** in the growth plate. PIIANP immunoreactivity was observed **(e)** around and within the lacunas of the inner zone, **(f)** in the superficial matrix of a section and **(g)** in the proliferating cells of the growth plate. **(h)** Preimmune serum control for CTX-II at the growth plate (PIIANP preimmune showed similar results, data not shown). Col IIA mRNA expression was observed **(i)** in the inner zone, **(j)** in the middle of a section showing the upper and deep zone, **(k)** in the proliferating chondrocytes of the growth plate. **(l)** Negative control using Col IIA sense probe. All sections were captured at ×20 magnification.

**Figure 5:**
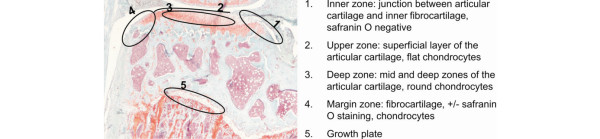
Description of the zones of interest for immunolocalisation and *in situ *hybridisation analysis. The picture shows a medial tibia plateau and surroundings including the underlying growth plate. The knee is separated into five zones, which are circled and described on the right. The inner zone of the tibia is defined as the area where the articular cartilage turns downwards into the space between the lateral and medial plateaus.

Second, we investigated whether CTX-II immunoreactivity was present, in which area of the medial tibia, and whether its presence related to the experimental condition. Figure 4(a) to 4(d) shows typical examples of how CTX-II immunoreactivity appears in the different areas of the cartilage, as defined in Figure 5. These include stainings immediately around chondrocytes as well as further away in the matrix and at the surface of areas where mechanical challenge is expected, but also away from such areas like in the inner zone and in the fibrocartilage of the margin zone.

**Figure 6:**
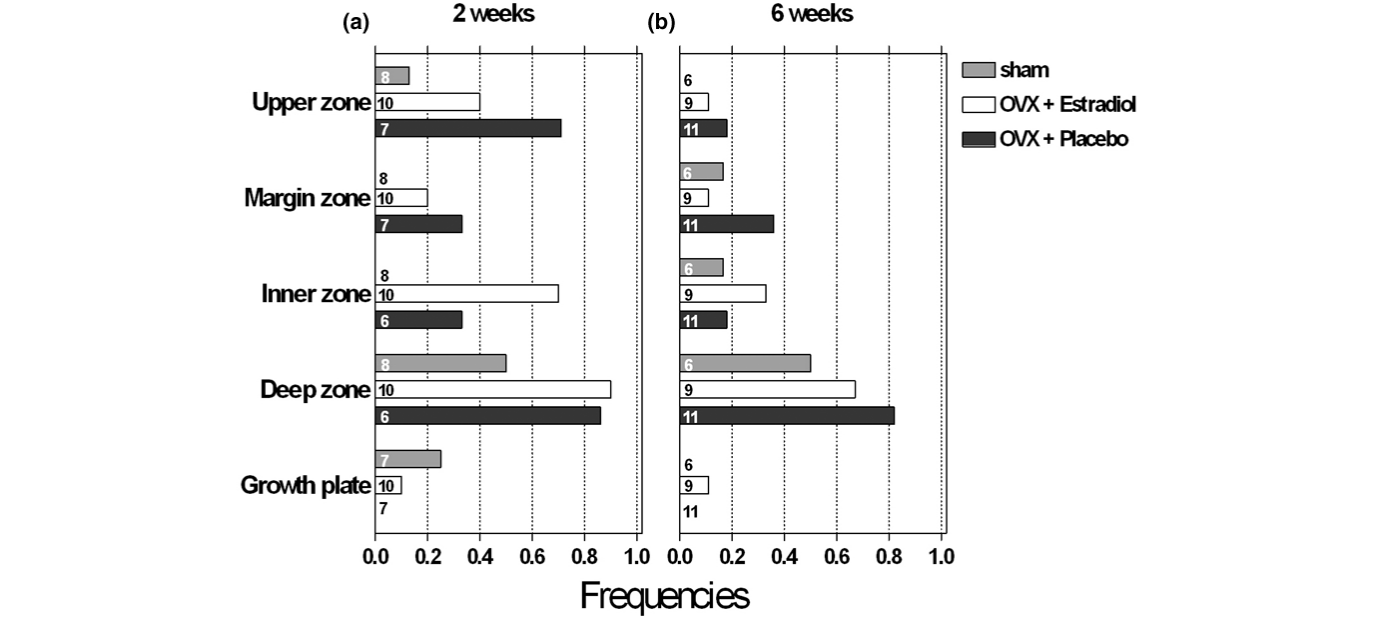
Effect of treatment on the frequency of CTX-II immunoreactivity within the zones of interest two and six weeks after ovariectomy. Numbers in each bar indicate the number of rats investigated.

In order to investigate the effect of oestrogen deprivation on CTX-II immunostaining, in each of these zones we analysed how frequently it was detected in the different rats (Figure 6). The cartilage of none of the rats from the two-week group showed CTX-II immunoreactivity at the margin and inner zone. Only a few showed signals in the upper zone and in the growth plate, but half of them showed signals in the deep zone (Figure 6a). Ovariectomised rats were more frequently positive in all these cartilage areas, except at the level of the growth plate. This increase in frequency was, however, smaller when these ovariectomised rats were treated with oestrogen, except in the inner and deep zones (Figure 6a). Overall, this analysis shows that it is only at the level of upper and marginal zones that the immunostainings reflected the pattern of urinary levels of CTX-II in the experimental groups. The cartilage of the rats from the six-week group tended to show less frequently CTX-II immunoreactivity compared with the two-week group, and the variations between the different experimental groups tended to become smaller, which is also reminiscent of behaviour of the urinary levels of CTX-II in these respective groups (Figure 6b). Despite the latter parallel seen when comparing the experimental groups, an analysis at the level of individual rats did not show significant correlations between urinary CTX-II and immunoreactivity in the tissue (e.g. at the upper zone p = 0.211 or at the deep zone p = 0.578).

**Figure 7:**
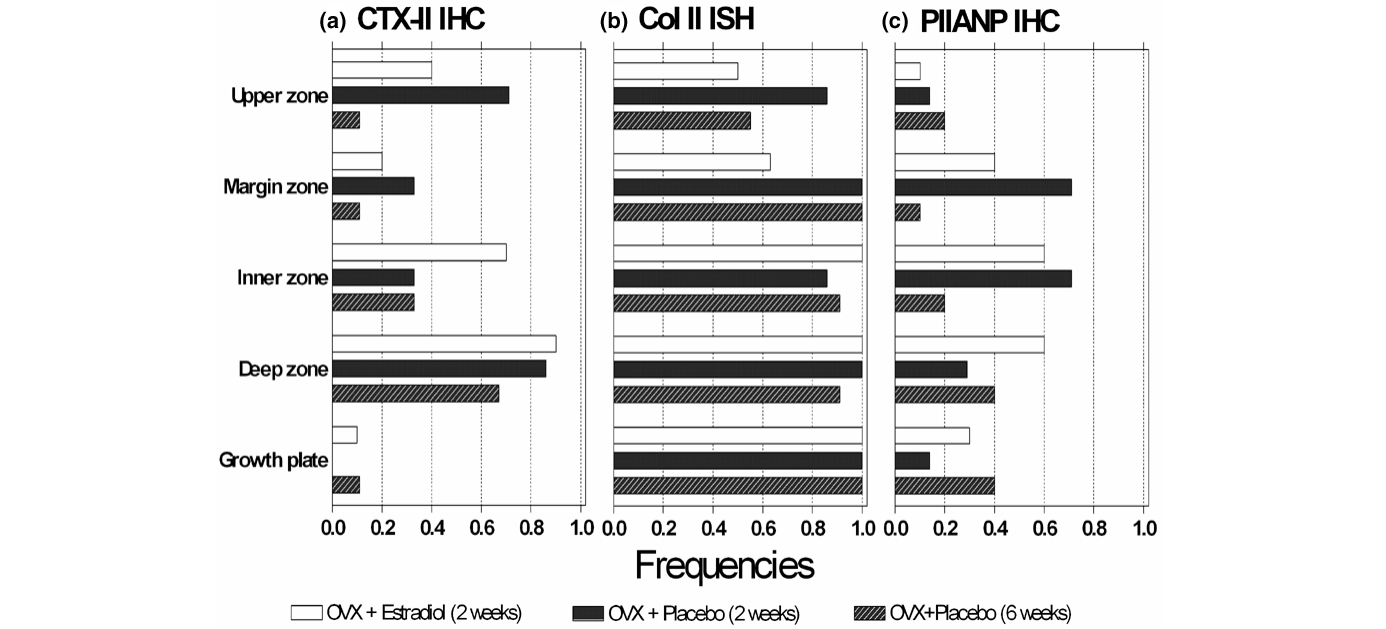
Effect of treatment on the frequency of type IIA collagen mRNA expression and PIIANP immunostaining compared with CTX-II immunostaining. Numbers in each bar indicate the number of rats investigated. **(a)** CTX-II immunohistochemistry (IHC); **(b)** type IIA collagen (Col II) *in situ *hybridisation (IHC); **(c)** PIIANP IHC.

It is not known whether CTX-II reflects degradation of pre-existing cartilage matrix, and/or of newly synthesised collagen. Thus, although the biochemical marker of collagen synthesis, PIIANP, did not indicate any influence of oestrogen deprivation, we also assessed collagen synthesis locally in cartilage tissue, both through PIIANP immunoreactivity (Figures 5e to 5h) and *in situ *hybridisation for type II collagen (Figures 5i to 5l), and investigated to what extent these signals would reflect CTX-II immunoreactivity in the tissue and correlate with the urinary levels of CTX-II (Figure 7). Type II collagen mRNA was found in all the cartilage areas of almost all the rats, and did not vary much according to the oestrogen status, except for the upper and margin areas, where it reflected the variations of CTX-II immunoreactivity in the different experimental conditions (Figure 7). Of these two zones, it is only in the upper zone that the mRNA showed a significant correlation with the urinary levels of CTX-II (p = 0.030 versus p = 0.943). PIIANP occurred less frequently compared with type II collagen mRNA, and was affected by oestrogen only at the level of the margin zone, which is also the only area where it parallels CTX-II (Figure 7). Our analysis did not indicate any significant correlation between PIIANP immunoreactivity in the tissue, whatever the zone, and urinary CTX-II (all zones; p > 0.05).

## Discussion

The hypothesis that oestrogen deficiency affects collagen turnover in cartilage, is supported to a large extent by measurements of a single marker, CTX-II, which responds to oestrogen status in a series of studies [16,17,26,32]. The present study demonstrates that body fluid levels of two other markers of type II collagen turnover did not respond to oestrogen deficiency, whether in ovariectomised rats and/or postmenopausal women. These other markers consisted in another type II collagen degradation marker, Helix-II [20], and a marker of collagen synthesis, PIIANP [22]. Interestingly, a recent report extends our observations to a third type II collagen degradation marker, C2C, which does not respond to oestrogen deficiency induced by menopause [33].

Situations where CTX-II shows a distinct behaviour compared with Helix-II and PIIANP have already been reported. For example, in OA all three markers are affected, but do not correlate strongly when analysed at the level of individual patients [20,24]. Immunostaining studies of OA cartilage have further established that to some degree they show differential selectivity for specific features into cartilage tissue [21]. Furthermore, it should be mentioned that CTX-II and Helix-II originate from the telopeptide and helicoidal domain of type II collagen, respectively, and that different proteinases were reported to be involved in their generation [34]. Therefore, the differences in behaviour between these two markers has been ascribed to histological or time-related differences in proteinase expression [21]. Overall, there are a series of situations where CTX-II behaves distinctly to other markers. The present study adds to these series the unique response of CTX-II to oestrogen deficiency.

There are many possible reasons why CTX-II is unique in its response to oestrogen deficiency. First, it may be speculated that oestrogen deprivation favours the proteolytic pathway generating CTX-II, compared with the one generating Helix-II [34]. The basis of this speculation is that oestrogens are known regulators of the proteinases that are critical for cartilage collagenolysis [35,36]. As a matter of fact, oestrogens were shown to determine the collagenolytic pathways used by osteoclasts to degrade bone [37,38]. Of note, C2C, the type II collagen degradation marker, which was recently reported to not respond to menopause, originates from the helix domain-like Helix-II [33].

Second, it has been proposed that distinct responses of markers to oestrogen reflect measurements of activities at different sites in the cartilage [33]. Our CTX-II immunostaining frequency analysis indeed indicates that specific areas of knee cartilage do not respond to oestrogen deficiency, whereas others do. Areas where CTX-II immunoreactivity did not respond to oestrogen status, but was frequent, included the deep and inner zone and growth plate, which is an area of high collagen turnover, and was previously considered to be a possible key contributor to urinary CTX-II [17]. It has been speculated that there is a relation between CTX-II and subchondral bone events [21,33]. The basis of this speculation was that both CTX-II and bone resorption are affected by oestrogen [16,26,32] and by a series of bone resorption inhibitors [18,39] and that the prevailing position in cartilage tissue of CTX-II is at the bone-cartilage interface [21]. However, the latter immunohistochemical study was performed in OA cartilage, and the present frequency analysis of CTX-II immunoreactivity in response to oestrogen status did not support this hypothesis, even if CTX-II was sometimes detected at this level as previously reported in rat knees [40].

In contrast, the present study shows that the CTX-II immunoreactivity response to oestrogen status in the upper and margin zone is similar to that of urinary CTX-II. This is compatible with a contribution of these zones to urinary CTX-II. Of note is that the upper zone is also the area where mild erosion appeared more frequently nine weeks after ovariectomy [17]. CTX-II immunoreactivity in this area was associated with erosion both in the present and in our earlier study [31]. However in the previous study, this CTX-II immunoreactivity detected nine weeks after ovariectomy was not analysed statistically. Therefore, it could not be related to ovariectomy-induced changes in urinary levels of CTX-II, because these changes occur only transiently and the CTX-II levels decreased to those of sham-operated rats at this nine-week time point. In order to relate oestrogen-induced changes in urinary levels of CTX-II to immunoreactivity frequencies at the cartilage level, our study was performed earlier after ovariectomy that is at the six-week time point. However, we did not obtain evidence for oestrogen-related lesions at these early time points, and rats showing immunoreactivity in the upper and margin zone were not necessarily those with high urinary CTX-II, questioning therefore to what extent this area contributes effectively to oestrogen changes in urinary CTX-II levels. Anyway, a limitation of these analyses is that they are restricted to knee cartilage when many other joints may contribute to CTX-II production in urine. As a matter of fact, the spine was proposed to be a major contributor in postmenopausal women [41,42]. In conclusion, our histological analysis of CTX-II indicates that the response of CTX-II to oestrogen status at the level of the medial tibia specifically concerns the upper and margin zone. The interesting question of whether this specific position differentiates CTX-II from Helix-II could not be assessed, because antibodies appropriate for immunohistochemistry of Helix-II in rat tissue were not available.

Our study relates the cartilage areas where CTX-II is detected most frequently with the areas where collagen synthesis occurs. It has been previously reported that not only collagen degradation, but also collagen synthesis is stimulated at least at some stage of OA [5]. It has been proposed that rapid degradation of newly synthesised collagen contributes to generating collagen fragments [43] and one may speculate that this degradation of newly synthesised collagen contributes unequally to the generation of CTX-II and Helix-II. Our study demonstrates active collagen synthesis whether evaluated through mRNA or PIIANP. Interestingly, the zones where synthesis responded to oestrogen were the upper and margin zones, like for the CTX-II immunostainings. However, they were probably too small to be reflected at the level of serum, and correlation studies at the level of individual rats did not support the hypothesis that oestrogen-induced changes in urinary levels of CTX-II originate from degradation of newly synthesised collagen.

**Figure 8:**
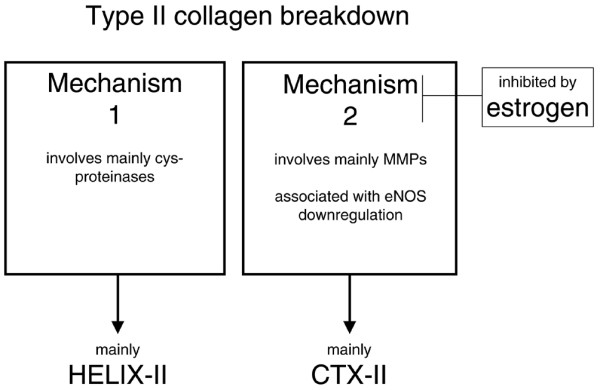
Selective effect of oestrogen on one of the two pathways for type II collagen breakdown. The evidence for two type II collagenolytic mechanisms, one related to cysteine proteinases and Helix-II, the other related to matrix metalloproteinase (MMPs) and CTX-II comes from Charni and colleagues [34]. The evidence for the selective effect of oestrogen on mechanism two comes from the present study. The evidence for the association of mechanism two with eNOS comes from Sniekers and colleagues [6], Grassi and colleagues [45] and Gurjar and colleagues [46].

Another important aspect of elevated urinary levels of CTX-II in response to oestrogen deprivation is that it is transient, because it returns to sham levels six to nine weeks after the ovariectomy, depending on the experiments [17,31]. The present study indicates the same tendency at the tissue level. A possible explanation for this is oestrogen production by adipose tissue, which acts locally in a paracrine/autocrine fashion, leading to locally high concentrations [44]. Ovariectomised rats increase their body weight with time compared with sham-operated rats, which means that the adipose tissue mass increases and therefore also possibly the release of oestrogen from adipose tissue increases. We speculate that the latter may compensate to some extent for the lack of ovarian oestrogen and attenuate progressively the elevation of CTX-II. However, oestrogen levels were below the detection limit, and therefore this hypothesis could not be verified. In addition, it might be of interest to investigate whether the transient ovariectomy-induced increase in CTX-II is related to ovariectomy-induced down-regulation of eNOS [45] (Figure 8). Indeed it is intriguing that both transient effects show similar kinetics, and that eNOS induces a decrease in matrix metalloproteinase activity [46], which are the proteinases responsible for CTX-II release [33]. Furthermore, oestrogen induces both an increase in eNOS and a decrease in MMP activity in chondrocytes [6].

## Conclusion

Our study demonstrates that oestrogen deficiency affects cartilage; however, only to a limited extent compared with what is seen in OA. We propose that oestrogen deficiency specifically affects the collagenolytic pathway that leads to a transient increase in urinary levels of CTX-II and later to mild alterations of the cartilage surface, but it does not significantly affect the pathway that generates Helix-II or C2C [33] (Figure 8). Although to some extent oestrogen affects collagen synthesis at the level of cartilage, it was not significantly reflected by changes in the urinary levels of PIIANP. Therefore, CTX-II appears as a marker able to detect early alterations after oestrogen deprivation that other markers cannot detect in a sensitive way.

## Abbreviations

Bp: base pair; CTX-II: C-terminal telopeptide of type II collagen; ELISA: enzyme-linked immunosorbant assay; H&E: haematoxylin and eosin; OA: osteoarthritis; PIIANP: propeptide of type IIA collagen; TBS: Tris buffered saline.

## Competing interests

The authors declare that they have no competing interests.

## Authors' contributions

ACBJ, TLA and JMD were the study directors, making protocols and the final analysis. All histology was performed by ACBJ. MK, FDH and LVS were responsible for the animal experiments and acted as scientific advisors. PG and NCBT measured biomarkers in the body fluids of humans and animals.
